# Prospective evaluation of a patented DNA test for canine hip dysplasia (CHD)

**DOI:** 10.1371/journal.pone.0182093

**Published:** 2017-08-03

**Authors:** Eberhard Manz, Bernd Tellhelm, Michael Krawczak

**Affiliations:** 1 Generatio Sol. GmbH, Veterinarian Institute of Molecular Genetics, Heidelberg, Germany; 2 Clinic for Small Animal Surgery, Justus-Liebig University, Gießen, Germany; 3 Institute of Medical Informatics and Statistics, Christian-Albrechts University, Kiel, Germany; University of Sydney Faculty of Veterinary Science, AUSTRALIA

## Abstract

Genetic testing has been propagated as a suitable means to specify individual risks for canine hip dysplasia (CHD). However, the current lack of validation of most genetic CHD tests has left dog owners and breeders in the dark about their practical utility. Therefore, the Society for German Shepherd Dogs (Verein für Deutsche Schäferhunde, SV) initiated a prospective study of 935 animals to assess independently the value of a genetic CHD test (European Patent Specification EP 2 123 777 B1) that was developed by Distl *et al*. (2009) on the basis of the SV animal stock. Dogs were followed-up for 3 years after birth, classified regarding their CHD phenotype using the scheme of the Fédération Cynologique Internationale, and genotyped for the 17 single nucleotide polymorphisms (SNPs) constituting the CHD test in question. Individual SNP genotypes were combined into animal-specific genomic breeding values (GBVs), calculated as the weighted sum of SNP-wise scores as laid down in the patent specification. Logistic regression analysis revealed that, unexpectedly, the odds ratio for CHD decreased, rather than increased, by a factor of 0.98 per unit increase of the GBV. Nevertheless, since this effect was not statistically significant (95% CI: 0.93–1.03), and the area-under-curve of the test was only 0.523, it must be concluded that the genetic test patented by Distl *et al*. (2009) is unsuitable for individual CHD risk assessment.

## Introduction

Traditional classification of canine hip dypslasia (CHD) is reliant upon phenotypic assessment, namely by radiography and Norberg-Angle measurement, of the hip joints of full-grown animals. Drawing upon the outcome of these examinations, most European breeding organizations have adopted a classification scheme established by the Fédération Cynologique Internationale (FCI) that specifies five grades of CHD severity (A-E). Class A corresponds to dogs with no signs of hip dysplasia whereas class E indicates presence of severe hip dysplasia. The classification process is laborious and costly but currently represents the only means to acquire useful information for animal selection aimed at reducing the incidence of CHD.

Breeding registers gather data from confirmed pedigrees and combine the CHD classification of individual animals with that of close relatives to yield so-called ‘estimated breeding values (EBVs)’. EBVs are specific to a phenotypic trait of interest and express the genetic merit of a given animal in units of that trait, relative to a certain base. For selection purposes, the absolute value of an EBV is usually not important but rather the differences in EBV between animals. An EBV-based selection program to combat CHD was started in 2002 by the Society for German Shepherd Dogs (Verein für Deutsche Schäferhunde, SV) for all their stud animals. Phenotype evaluation has been mandatory in the program if studbook-documented breeding was aspired to, and only dogs rated grade A to C got unrestricted breeding permission. Among the dogs enlisted in the EBV-based selection program, the prevalence of CHD class A animals had increased from 62% to 75% by the end of 2016.

Various attempts have been made to identify genetic loci underlying CHD development with the goal to develop a DNA test that would outperform, and eventually obviate, phenotype-based selection for CHD. Thus, Guo *et al*. [1] devised a genomic breeding value (GBV) that was similar to approaches taken in livestock breeding programs before. A learning cohort of 359 animals was characterized for CHD by way of traditional EBVs and genotyped for some 22,000 single nucleotide polymorphisms (SNPs). Based upon the SNP allele distribution and the available EBVs, a GBV formula was developed that resulted in 70% to 90% correlation between GBVs and EBVs. For validation, the formula was also applied to 38 dogs previously classified for CHD and typed at roughly 13,000 SNPs [1]. The resulting GBVs showed moderate correlation (around 50%) with CHD class, and the sensitivity, specificity, positive and negative predictive values of the GBVs all lay in the range of 70% to 75%. Notably, the correlation between GBV and EBV remained virtually unchanged when the calculations were confined to the most informative 100 to 500 SNPs. To increase CHD selection response, the authors suggested that EBV be replaced by GBV, but at the same time emphasized that progeny phenotyping and EBV calculation should continue so as to allow improvement (i.e. retraining) of the GBV formula, if required.

A different approach to CHD prediction was taken by Distl *et al*. [2]. Their assessment of genetic CHD predisposition relied upon only 17 polymorphisms (16 SNPs, 1 indel) previously reported as being associated with CHD in German Shepherd Dogs [3]. According to the patent (European Patent Specification EP 2 123 777 B1) filed for the marker set and for the algorithm to compute a GBV from animal-specific marker genotypes, each marker is indicative of the genetic disposition for CHD. Homozygous wild-type genotypes were ascribed a positive (beneficial) effect while homozygous mutant genotypes were ascribed a negative (deleterious) effect. Heterozygous animals are predisposed according to a marker-specific dominance effect that usually places them somewhere in between the two homozygous states. The contribution of each individual marker in the form of numerical weights is part of the patent claim. Summation of the marker-specific weights yields the GBV of a given animal which is supposed to reflect its tendency to develop and pass on CHD. The individual numerical CHD risk is determined by gauging the GBV against a reference curve. The inventors also alluded to the possibility of predicting the risk of CHD in the offspring of genotyped dogs (section [0022]) but did not make this an explicit part of patent claim. Notably, the patent specification does not include any information about a possible validation of the GBV and, to the best of our knowledge, no such validation has been reported in the scientific literature.

A procedure similar to that proposed by Distl *et al*. [2], but targeted at Labrador Retrievers, was recently devised by Bartolomé *et al*. [4] and a patent was filed for this procedure as well (EP 2619319 A2). Prediction of CHD in Labrador Retrievers is based upon only seven SNPs that were identified in the course of a genome-wide association study with the Illumina canine HD BeadChip. SNP genotypes served as independent variables in a standard logistic regression analysis, and the final regression model reportedly achieved good predictive capability in an independent validation cohort. The area under curve equaled 0.8 whilst the combination of sensitivity and specificity ranged from 93%/58% via 80%/78% to 42%/95%. The good performance of the test was attributed to the fact that the seven SNPs were closely linked to genes that are thought to directly influence hip joint development. The authors also announced further validation of the test in Labradors and other breeds.

Despite the fact that the marker set and GBV algorithm proposed for German Shepherd Dogs by Distl *et al*. [2] obviously have never been validated, the procedure was proposed as the long sought-after DNA-based CHD test for this breed. Therefore, the Verein für Deutsche Schäferhunde e.V. (German Society for German Shepherd Dogs, SV) initiated a prospective study among their members to systematically investigate the validity of the claims made in connection with patent EP 2 123 777 B1 [2]. The study was carried out between 2012 and 2015 under the auspices of the SV. Here, we report on the design, results and implications of these investigations. CHD is morphologically similar to another detrimental bone condition, namely canine elbow dysplasia (CED). Therefore, an explorative evaluation of the 'predictive capability' of the investigated GBVs for CED was attached to the CHD study as a negative control not hitherto linked to the SNPs in question.

The present study was devised to validate the patented marker set and GBVs according to their intended use in individual dogs, as laid down in the patent claims. An originally planned multi-generational follow-up of the included dogs was abandoned as a consequence of the poor performance of the test in the present study. In fact, section [0022] of the patent contains a description of how to use the markers and GBVs to predict CHD in the prospective offspring of pairs of genotyped dogs as well. To this end, the patent proposes calculation of the probabilities of all possible offspring genotypes, assuming Mendelian inheritance, followed by subjecting the potential offspring genotypes to the same CHD risk assessment as described for experimentally determined genotypes. The weighted average of these genotype-specific risks is then taken as the overall CHD risk of prospective offspring. Since the same method of CHD risk assessment is used in both instances, however, the feasibility of risk calculation for prospective offspring would require, at the very least, the feasibility of risk calculation for singletons.

## Materials and methods

### Animals

Over a period of three years (2012–2015), a total of 1110 purebred German Shepherd Dogs from the SV breeding population were included in the study. To qualify for participation, dogs had to have a starting age of 12 to 15 weeks at that time of inclusion, and the dog owner had to commit themselves to filling out a monthly questionnaire until the animal reached 15 months of age. A maximum of two dogs per owner were included to ensure population representativeness. Animals were radiographed by SV-approved veterinarians when the dog was 12 months or older, and a blood sample was taken. The timing of these examinations was exactly as in the original association study that led to both, the identification of the patented marker set and the development of the GBVs [3]. Radiographs were evaluated according to the FCI scheme by one of us (BT), acting as expert veterinarian on behalf of the SV. Note that FCI grades A to E correspond to phenotypic classes 1 to 5 of the SV classification scheme, which was used for CHD phenotyping in the present study. Blood samples were stabilised by EDTA and stored for further analysis after the data collection period.

#### Ethics statement

By drawing upon data that would have been generated anyway, the study aimed at avoiding any unnecessary interference with the well-being of animals. SV members were informed about the study and its design through SV-internal media. Participation in the study was on a strictly voluntary basis and consent was obtained for any necessary processing of personal data (i.e. for study registration and filling-out of the online questionnaires). All animal work, including immobilization, radiography of hip joints and blood sample collection for DNA typing was part of the regular CHD/CED assessment for breeding capability and was not carried out for the purpose of the study. All animal treatment was conducted by trained veterinarians from an SV-accredited pool of experts. This approach obviated the need for explicit ethics committee approval.

### Markers

PCR primers for the amplification of 17 CHD-associated markers were designed according to the specification of patent EP 2 123 777 B1 (S2 Table). However, allelic and flanking DNA sequence information was adopted only if it could be confirmed experimentally in our own laboratory. In fact, it turned out that the DNA sequence at four of the markers (TiHo1, TiHo18, TiHo19 and TiHo35) had to be revised because the information given in the patent specification was wrong (see Table 1 for the necessary adjustments).

**Table 1:** Revised DNA sequence context of four CHD markers (based upon own experiments).

Marker	Wild-type Sequence	CHD-associated Mutant Sequence
TiHo1a	CAAGAGT[A]TCCAGTTCC	CAAGAGT[G]TCCAGTTCC
TiHo18	TTCCCTCCCTGTG[T]TTCCTTCCA	TTCCCTCCCTGTG[G]TTCCTTCCA
TiHo19	CTAAAATCTGA[C]ATAGCCAAAG	CTAAAATCTGA[T]ATAGCCAAAG
TiHo35	TTAGAAAGGT[G]ACTTTCCAGG	TTAGAAAGGT[C]ACTTTCCAGG

### DNA isolation and genotyping

DNA was isolated from EDTA blood samples using the nexttec^™^ 1^-Step^ DNA Isolation Systems kit (nexttec, Hilgertshausen, Germany) according to the manufacturer’s protocol. The identity of each DNA sample was verified in the course of the routine SV program for DNA profiling and paternity analysis. Purified DNA was SNP-genotyped using KASP^™^ technology (LGC, Hoddesdon, UK). For each of the 17 CHD-associated SNPs, DNA was added to a KASP assay mix containing two allele-specific primers and a common reverse primer. Sample DNA was stored in 96 well master plates and daughter plates were prepared for each marker for genotyping. KASP assay master mix was added by automated pipetting.

Prior to its use in the present study, each KASP assay was tested for its genotyping accuracy by a comparison of assay-derived genotypes with the results of direct Sanger sequencing. To this end, controls were included on each plate that had been genotyped for all CHD markers before by Sanger sequencing. To bolster the trustworthiness of KASP typing even further, a mutation in the SOD-1 gene associated with degenerative myelopathy [5] was also included in the genotyping efforts. All animals from the present study had been genotyped for this mutation before by Sanger sequencing in a project to investigate its frequency in the German Shepherd Dog population managed by SV (manuscript in preparation). Consistency of the SOD-1 mutation genotypes, in combination with automated pipetting, generated strong confidence in the authenticity of the individual CHD marker genotypes. An Excel file containing all CHD marker genotypes alongside the corresponding CHD and CED classifications is provided as S1 Table.

### Computation of genomic breeding values GBV and GBV(S)

All animals in our study were genotyped for the 17 SNPs listed in Table 1 and S2 Table. In accordance with section [0043] of the patent specification, each animal was assigned the marker-specific numerical weight given in S3 Table if they were found to be homozygous or heterozygous for the CHD-associated (mutant) allele of the marker in question. Animals homozygous for the wild-type allele were assigned the negative of the entry in column ‘homozygous’ of S3 Table. Finally, marker-specific numerical weights were summed up to yield an overall genomic breeding value GBV according to section [0022] (see S1 Text for the dBASE script used). This value was further standardized as laid down in section [0025] of the patent specification (i.e. subtraction of the study-wide mean followed by division by the study-wide range of GBV). Since the standardized value was mistakably referred to as a ‘standard deviation’ in the patent specification, we chose to abbreviate this quantity as GBV(S) rather than ‘dev’ as originally proposed.

### Statistical analysis

Differences in terms of GBV or GBV(S) between different phenotypic groups were assessed for statistical significance using a Kruskal-Wallis test as implemented in SAS 9.4 procedure NPAR1WAY (SAS Institute Inc., Cary, NC, USA). Odds ratios (OR) and relative risks (with 95% confidence intervals) were estimated using the MedCalc software (www.medcalc.org). Logistic regression analysis was carried out, and the area-under-curve (AUC) determined for predictive models, with SAS procedure LOGISTIC. Box plots were prepared with SigmaPlot 8.02 (SPSS Inc., Chicago, IL, USA).

All data used in this study, including individual marker genotypes, derived GBVs as well as individual CHD and ED phenotypes are available from the authors upon request.

## Results

### Technical marker validation

All marker locations as given in the description of patent EP 2 123 777 B1 could be confirmed (S2 Table). For a few markers, however, some other characteristics were incorrect and had to be revised (Table 1).

#### TiHo1

The CHD-associated allele was originally reported to be a G insertion between two T residues, i.e. CAAGAGT[G]TCCAGTTCC. In our own sequencing experiments, however, the implied wild-type allele lacking G was never observed. Instead, either an A or a G was present at the respective position so that the marker seems to constitute a single base-pair substitution rather than a one base-pair indel. This finding was further supported by reference to the CanFam3.1 assembly, where CAAGAGT[G]TCCAGTTCC is also logged at the respective position. We therefore suggest revision of SNP TiHo1 to CAAGAGT[A/G]TCCAGTTCC.

#### TiHo18

In the patent description, both sequences of this SNP were given in reverse direction relative to CanFam3.1, thereby specifying an A/C SNP with C as the CHD-associated allele. Transformation from lagging to leading strand was thus carried out to comply with the reference assembly and, in consequence, SNP TiHo18 now constitutes a T/G exchange. The CHD-associated allele is G instead of C.

#### TiHo19

The wild-type sequence in the patent description ([2], table 1) is identical to the mutant sequence description ([2], table 2). According to the patent description ([2], table 3) the SNP is a C/T substitution with C being the CHD-associated mutation.

#### TiHo35

The wild-type sequence in the patent description ([2], table 1) is identical to the mutant sequence description ([2], table 2). According to the patent description ([2], table 3), the SNP is a C/G substitution with G being the CHD-associated mutation.

### CHD and CED status

A total of 935 dogs were assessed in terms of their CHD status (Table 2). For 928 of these, diagnostic information was also available for CED. Most animals were affected by neither disease (phenotype class 1). More specifically, 657 dogs (70.3%) were free of CHD whilst 714 (76.9%) lacked signs of CED. Of the 287 animals suffering from CHD, 179 (62.4%) showed a mild phenotype (class 2) whereas 25 (8.7%) were diagnosed with severe CHD (class 5). The remainder had a mild to moderate phenotype (class 3: 53, 18.5%; class 4: 21, 7.3%). In the case of CED, 70 of 214 affected dogs (32.7%) suffered from a mild form (class 2) whereas 37 (17.3%) had severe CED (class 5). Some 39 (18.2%) and 68 (31.8%) animals were diagnosed with mild to moderate CED (class 3 or 4, respectively). The two conditions were found to be significantly associated with one another in that 525 dogs (56.6%) had neither condition whilst 82 (8.8%) had both (OR: 1.73, 95% CI: 1.25–2.38).

**Table 2:** Distribution of canine hip dysplasia (CHD) and elbow dysplasia (CED).

Disease	Phenotypic Class					Total
	1	2	3	4	5	
CHD	657	179	53	21	25	935
CED	714	70	39	68	37	928

For both diseases, unaffected animals were assigned phenotypic class 1.

### Phenotype prediction

As was to be expected owing to the order-preserving nature of standardization transformations, no notable differences in terms of GBV or GBV(S) were observed between affected and non-affected animals for either disease. Thus, the median GBV value was equal to 1.018 (inter-quartile range IQR: 4.055) among dogs diagnosed with CHD, compared to 1.442 (IQR: 3.346) in the unaffected group. Contrary to expectation, the genomic breeding value GBV, which is supposed to be positively correlated with CHD risk, was therefore decreased rather than increased in affected animals, although the difference between the two groups was not statistically significant (Kruskal-Wallis χ^2^ = 1.170, 1 d.f., p>0.2). Virtually identical results were obtained for GBV(S) (data not shown). In the case of CED, the results were also negative as expected in that the median GBV was 1.378 (IQR: 3.090) for affected and 1.320 (IQR: 3.670) for unaffected animals (Kruskal-Wallis χ^2^ = 0.970, 1 d.f., p>0.3).

When the predictive utility of GBV for CHD was assessed by way of logistic regression analysis, an OR increase per unit GBV increase of 0.98 emerged that was not however significantly different from unity (95% CI: 0.93–1.03). Moreover, the AUC of the predictive test based upon the logistic-regression-derived CHD risk amounted to a minuscule 0.523.

**Fig 1:**
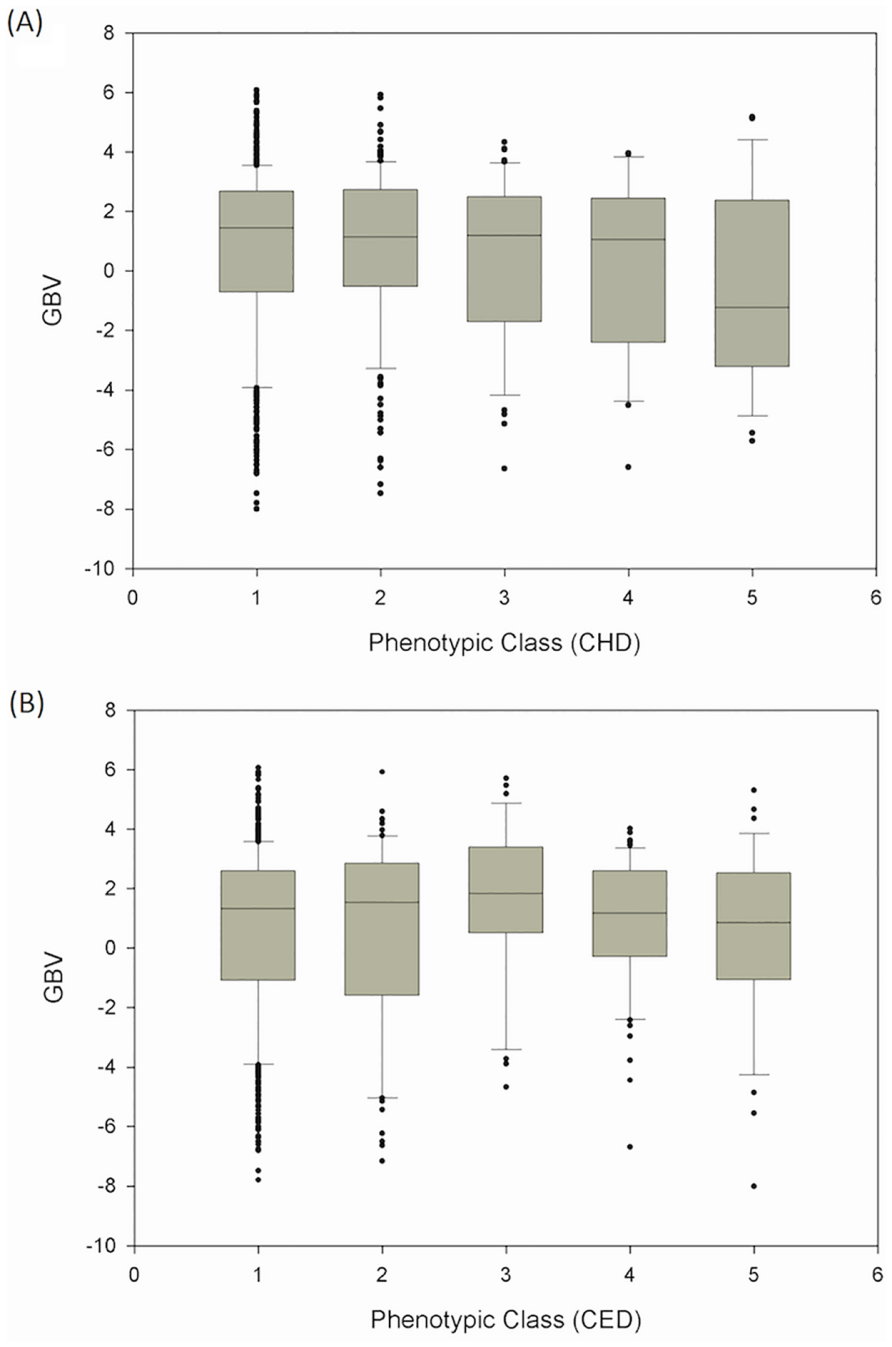
Relationship between genomic breeding value GBV and severity (phenotypic class) of CHD (Fig 1A) and CED (Fig 1B).

Upon closer inspection of the relationship between the phenotypic classification and GBV, a notably negative trend became apparent for CHD (Fig 1A), but not for CED (Fig 1B). With increasing CHD classification (from class 1 to class 5), the median GBV decreased from 1.442 via 1.134 (classes 2, 3 and 4 combined) to -1.234 (class 5). If anything, GBV as proposed in patent specification EP 2 123 777 B1 therefore provides a predictive ‘test’ of minor utility (*post hoc* AUC: 0.622) for severe CHD, but only after inverting the interpretation of the test result.

In view of the poor performance of GBV and GBV(S), we tried to develop a post hoc predictive model for CHD, based upon the genotypes available for the 17 SNPs in question. To this end, each marker genotype was encoded by a combination of two variables, namely the dosage of the respective CHD-associated allele (0, 1 or 2) and a covariate for the dominance effect equal to 1 for heterozygotes and 0 for homozygotes.

Even the full predictive model including all additive and dominance effects of the 17 markers yielded only minor predictive utility for CHD (AUC: 0.610). Stepwise selection of predictors as implemented in SAS procedure LOGISTIC, and employing a significance threshold of 5%, the final model comprised only the allele dosage of markers TiHo25 (score χ^2^ = 5.794, 1 d.f., p = 0.016) and TiHo26 (score χ^2^ = 5.865, 1 d.f., p = 0.015). The estimated OR per HD-associated allele was 0.78 (95%CI: 0.61–0.95) for TiHo25 (allele G) and 1.41 (95% CI: 1.14–1.75) for TiHo26 (allele C). No other significant additive or dominance effects were observed. The *post hoc* predictive utility of the final model was however found to be poor (AUC = 0.567).

An analogous analysis carried out for severe CHD (class 5) also included the allele dosage of TiHo25 in the final model (score χ^2^ = 6.525, 1 d.f., p = 0.011), with an estimated per-allele OR of 0.54 (95%: 0.33–0.88). In addition, two highly significant dominance effects were observed, namely for TiHo12 (score χ^2^ = 17.614, 1 d.f., p<0.0001) and for TiHo18 (score χ^2^ = 7.249, 1 d.f., p = 0.007). Taken together, the three genetic risk factors yielded moderate to high predictive utility (*post hoc* AUC: 0.793). The two dominance effects were indeed remarkable. Whilst none of the 21 wild-type homozygotes for TiHo12 and only 9 of the 660 homozygotes for CHD-associated allele T developed severe CHD, this was true for 16 of 254 heterozygotes. Considered *post hoc*, this would imply a relative CHD risk of 4.77 (95% CI: 2.13–10.65) for genotype TC. For GC heterozygotes at marker TiHo18, a relative CHD risk of 2.94 (95% CI: 1.28–6.75) became apparent.

## Discussion

The genetic architecture of complex diseases like CHD differs fundamentally from that of monogenic disorders. Whereas the latter, by definition, are due to a few genetic changes with high penetrance, the former result from the interplay of a large and unknown number of environmental and genetic factors, most of which have small effects [6]. Nevertheless, given the rapid technological development in genetics research, it can be expected that the vast majority of genetic loci contributing appreciably to a complex disease, in humans as well as in non-human mammals cared for by humans, will be identified in the not too distant future [6]. Even though unraveling statistical genotype-phenotype relationships is only a first step towards a better understanding of disease etiology, such associations can nevertheless be of immediate practical benefit in that they may form the basis of diagnostic or prognostic tests. This includes the prediction of canine diseases such as CHD and CED.

A number of CHD-associated genetic loci have been identified in the past, and the 17-marker panel proposed by Distl *et al*. [2], for which a European patent (EP 2 123 777 B1) was filed in 2009, has been promoted as the most promising candidate for a predictive test for CHD so far. However, to the best of our knowledge, this panel has never been validated in an independent animal population. Furthermore, no studies of the practical applicability and utility of the patent claims have been published in the scientific literature so far. Remarkably, the same group has published other genetic markers of CHD [7,8], but the only reference to the patent made in these articles was mentioning Marschall & Distl [3] in the course of introducing the literature on CHD-associated markers. The patented markers themselves were not experimentally investigated further.

Under the auspices of the German Society for German Shepherd Dogs (SV), a prognostic study was therefore performed to investigate the predictive capability of the patented SNP panel for CHD in a large, independent and population-representative sample. The primary intention of the study was to assess the capability of the SNPs to predict individual CHD development. An extension covering the prediction of CHD in the offspring of genotyped dogs was scheduled for after successful completion of the first study phase, but was later abandoned.

In addition to the technicalities of the CHD-associated SNPs involved, the patent specification also contains instructions for calculating a standardized genomic breeding value ‘dev’ (here abbreviated as GBV(S)) from a given SNP profile, which in turn can be used to quantify the individual risk of developing CHD by gauging GBV(S) against a reference curve that was also included in the patent description. According to the instructions given, GBV(S) is expected to range from—0.5, reflecting the most beneficial genetic disposition, to +0.5 for animals at the highest disease risk. Such a range would result if the non-standardized breeding value GBV followed a symmetrical distribution. In our study, by contrast, GBV(S) was found to range from -0.613 to +0.387 owing to the pronounced skewness of GBV as observed in the data. Most importantly, however, neither GBV nor GBV(S) was found to bear any appreciable statistical relationship to phenotypic CHD.

The utility of a predictive test with a continuous outcome such as, for example, GBV or GBV(S) can be assessed by way of the so-called ‘receiver-operating characteristic curve’ (ROC). The ROC relates the probability of true positive results to the probability of false positive results that would ensue from using distinct thresholds to dichotomize the test outcome. The AUC sensibly ranges from 0.5 (equivalent to tossing a coin, i.e. total lack of predictive capability) to 1.0 (perfect prediction) and is independent of the prevalence of the disease in question. According to Janssens *et al*. [9], an AUC of around 0.8 is usually regarded as both necessary and sufficient for screening purposes. However, the genomic breeding value GBV proposed by Distl *et al*. [2] achieved an AUC of only 0.523 for CHD in our study. Since this rather poor performance, in principle, could have reflected inadequacy of the marker weights used for the definition of GBV, we subjected the SNP genotypes to post hoc logistic regression analysis, allowing for all possible additive and dominance effects. Nevertheless, even the full regression model, including all 17 markers, yielded an AUC of just 0.610 in our study. Moreover, this result was found to hinge essentially on just two markers, namely TiHo25 and TiHo26. All other markers lacked even minuscule predictive value for CHD. Notably, when applied to CED, the 17 SNPs also lacked any appreciable predictive value as was to be expected because CED served as a kind of negative control in the present study.

With hindsight, our results may not have been surprising because, despite the uncontested prospects of genomic selection in livestock and plant breeding [10], the vast majority of genotype-phenotype relationships in complex diseases studied so far in humans as well as in non-human species have turned out to be of little, if any, diagnostic or prognostic value [11]. This is why independent validation in representative population samples should be a *conditio sine qua non* of any practical use of tests exploiting such relationships. While emphasizing their potential usefulness, Wray et al. [10] also highlighted the necessity of prior validation of SNP-based genomic breeding values before they can be sensibly applied for selection purposes in the context of complex traits. If this advice had been followed in the present case, the poor performance of the CHD test proposed by Distl et al. [2] would have become apparent well before its attempted commercialization.

Instead of basing CHD prediction upon a small number of pre-selected markers, Guo *et al*. [1] proposed the inclusion of all available genomic information in a prognostic model, an approach that recalls strategies in livestock and crop breeding programs for quantitative traits alluded to above. In their ‘agnostic’ approach, all SNPs on a given chip that turn out disease-associated in a ‘learning population’ are eventually included in the CHD risk calculated from the genetic profiles of other animals. Interestingly, Guo et al. [1] also suggested constant updating of the respective SNP panel on the basis of subsequently acquired phenotypic and CHD-specific genetic information. This approach would clearly contradict the validation requirement emphasized by Wray et al. [10] and therefore highlights the difficulties in finding a consensus on how to genetically predict complex traits.

## Conclusions

In a three years prospective study of nearly 1000 animals, we have evaluated and shown invalid a prognostic, SNP-based test for canine hip dysplasia (CHD) that was patented [2] based upon data from a previous scientific publication [3]. The test has since been propagated to various potential clients as a suitable means of animal selection in practical breeding, including the German Society for German Shepherd Dogs (SV) who acted as main sponsor of the present study. In its original form, the SNP-based test turned out to be completely incapable of predicting CHD in our target population. If anything, some of the respective SNP may be predictive of severe CHD, but such a combined test would require validation in another independent study to be credible. For the time being, practical application of the SNP-based test proposed for the prediction of CHD in German Shepherd Dogs is thus not recommended.

Our assessment of an alleged genetic test for CHD is the first systematic and independent attempt to validate a DNA-based predictive procedure meant for practical use in a non-human mammal species. In this respect, we regard our work as exemplary for the caution required towards any genetic test claimed to predict complex multifactorial traits in the field. Whatever evidence apparently underpins its predictive capability in the original report, a predictive test should be considered fit for practical use only after it has been tested successfully and independently in the intended target population.

## Supporting information

S1 Table Excel file comprising CHD and CED classification values together with individual CHD marker genotypes. (XLSX)

S2 Table DNA sequences of 17 CHD-associated genetic markers (according to tables 1 and 2 of patent EP 2 123 777 B1). (DOCX)

S3 Table CHD markers and associated numerical weights (according to table 6 of European Patent Specification EP 2 123 777 B1). (DOCX)

S1 Text dBASE program used for the calculation of GBVs from SNP genotypes. (PDF)

## References

[1] Guo G, Zhou Z, Wang Y, Zhao K, Zhu L, Lust G, et al Canine hip dysplasia is predictable by genotyping. Osteoarthritis Cartilage 2011 19(4), 420–429. doi: 10.1016/j.joca.2010.12.011 21215318 10.1016/j.joca.2010.12.011 PMC3065507

[2] Distl O, Marschall Y, Stock KF, inventor. Analysis for the genetic disposition for hip dysplasia in Canidae. EP 2123777 B1 European Patent Office Bulletin 2013/52, p. 672

[3] Marschall Y, Distl O. Mapping quantitative trait loci for canine hip dysplasia in German shepherd dogs. Mamm Genome. 2007 12;18(12):861–70. doi: 10.1007/s00335-007-9071-z Epub 2007 Nov 20. 18027024 10.1007/s00335-007-9071-z

[4] Bartolomé N, Segarra S, Artieda M, Francino O, Sánchez E, Szczypiorska M, et al A genetic predictive model for canine hip dysplasia: Integration of genome wide association study (GWAS) and candidate gene approaches. PLoS One. 2015 4 13;10(4). doi: 10.1371/journal.pone.0122558 eCollection 2015. 25874693 10.1371/journal.pone.0122558 PMC4395148

[5] Awano T, Johnson GS, Wade CM, Katz ML, Johnson GC, Taylor JF, et al Genome-wide association analysis reveals a SOD1 mutation in canine degenerative myelopathy that resembles amyotrophic lateral sclerosis. Proc Natl Acad Sci USA. 2009 2 24;106(8):2794–9. doi: 10.1073/pnas.0812297106 Epub 2009 Feb 2. 19188595 10.1073/pnas.0812297106 PMC2634802

[6] Antonarakis SE, Chakravarti A, Cohen JC, Hardy J. Mendelian disorders and multifactorial traits: the big divide or one for all? Nat Rev Genet. 2010 5; 11(5):380–4. doi: 10.1038/nrg2793 20395971 10.1038/nrg2793

[7] Fels L, Distl O. Identification and validation of quantitative trait loci (QTL) for canine hip dysplasia (CHD) in German shepherd dogs. PLoS One. 2014 5 6;9(5) doi: 10.1371/journal.pone.0096618 24802516 10.1371/journal.pone.0096618 PMC4011879

[8] Fels L, Marschall Y, Philipp U, Distl O. Multiple loci associated with canine hip dysplasia (CHD) in German shepherd dogs. Mamm Genome. 2014 6;25(5–6):262–9. doi: 10.1007/s00335-014-9507-1 Epub 2014 Apr 2. 24691653 10.1007/s00335-014-9507-1

[9] Janssens AC, Aulchenko YS, Elefante S, Borsboom GJ, Steyerberg EW, van Duijn CM. Predictive testing for complex diseases using multiple genes: fact or fiction? Genet Med. 2006 7;8(7):395–400. 16845271 10.1097/01.gim.0000229689.18263.f4

[10] Wray NR, Yang J, Hayes BJ, Price AL, Goddard ME, Visscher PM. Pitfalls of predicting complex traits from SNPs. Nat Rev Genet. 2013 7;14(7):507–15. doi: 10.1038/nrg3457 Epub 2013 Nov 18. 23774735 10.1038/nrg3457 PMC4096801

[11] Makowsky R, Pajewski NM, Klimentidis YC, Vazquez AI, Duarte CW, Allison DB, et al Beyond missing heritability: Prediction of complex traits. PLoS Genet. 2011 4;7(4). doi: 10.1371/journal.pgen.1002051 Epub 2011 Apr 28. 21552331 10.1371/journal.pgen.1002051 PMC3084207

