# Supplementary material for: Prospective evaluation of a patented DNA test for canine hip dysplasia (CHD)
Source: PLoS One. 2017 Aug 3;12(8):e0182093. doi: 10.1371/journal.pone.0182093 (PMC5542656; doi:10.1371/journal.pone.0182093)
Supplement: S2 Table — (DOCX) [file pone.0182093.s002.docx]

**S2 Table:** DNA sequences of 17 CHD-associated genetic markers (according to tables 1 and 2 of patent EP 2 123 777 B1)

| **Marker** | **Wild-type Sequence** | **CHD-associated Mutant Sequence** |
| --- | --- | --- |
| TiHo1 | CAAGAGT[]TCCAGTTCC | CAAGAGT[G]TCCAGTTCC |
| TiHo1a | CAAGAGT[A]TCCAGTTCC |  |
| TiHo5 | GCAATGCAT[C]GGTTGTTTTT | GCAATGCAT[T]GGTTGTTTTT |
| TiHo7 | ACCTTAGGTA[G]TACCAAATA | ACCTTAGGTA[T]TACCAAATA |
| TiHo9 | TAAGAATGA[G]AGTGTATTTTGTC | TAAGAATGA[T]AGTGTATTTTGTC |
| TiHo12 | CAACATTGTTA[C]ACTAAACACTG | CAACATTGTTA[T]ACTAAACACTG |
| TiHo16 | AATTCTCAC[C]GAAAGTCTGCCAG | AATTCTCAC[T]GAAAGTCTGCCAG |
| TiHo18 | TGGAAGGAA[A]CACAGGAGGGAA | TGGAAGGAA[C]CACAGGAGGGAA |
| TiHo19 | CTAAAATCTGA[C*]ATAGCCAAAG | CTAAAATCTGA[C*]ATAGCCAAAG |
| TiHo20 | GTACTTGGA[T]GCTGCATAC | GTACTTGGA[C]GCTGCATAC |
| TiHo21 | CAAACACGTGA[T]GTCTTTAAA | CAAACACGTGA[C]GTCTTTAAA |
| TiHo23 | AGGAAGGACAG[T]GCCTTGCCCT | AGGAAGGACAG[C}GCCTTGCCCT |
| TiHo24 | GTGGCAGAT[A]TGAGTCAC | GTGGCAGAT[G]TGAGTCAC |
| TiHo25 | CAGGATGGC[A]CCCCAGTTC | CAGGATGGC[G]CCCCAGTTC |
| TiHo26 | TAGCTTCCTG[A]AATACCATTAT | TAGCTTCCTG[C]AATACCATTAT |
| TiHo33 | CTTACCTGC[G]TCCCTTCCCC | CTTACCTGC[A]TCCCTTCCCC |
| TiHo34 | TGTGAGTT[A]AACATGTAAAA | TGTGAGTT[G]AACATGTAAAA |
| TiHo35 | TTAGAAAGGT[G*]ACTTTCCAGG | TTAGAAAGGT[G*]ACTTTCCAGG |

* The SNP was incorrectly logged with two identical alleles in the patent description.
