# Supplementary material for: Prospective evaluation of a patented DNA test for canine hip dysplasia (CHD)
Source: PLoS One. 2017 Aug 3;12(8):e0182093. doi: 10.1371/journal.pone.0182093 (PMC5542656; doi:10.1371/journal.pone.0182093)
Supplement: S3 Table — (DOCX) [file pone.0182093.s003.docx]

**S3 Table:** CHD markers and associated numerical weights (according to table 6 of European Patent Specification EP 2 127 777 B1)

| Marker | CHD allele | Wild-type  allele | Numerical Weight | |
| --- | --- | --- | --- | --- |
|  |  |  | Homozygous | Heterozygous |
| TiHo01a | G | A | +0.459 | +0.074 |
| TiHo05 | T | C | +0.477 | -0.036 |
| TiHo07 | T | G | +0.327 | +0.019 |
| TiHo09 | T | G | +0.391 | +0.016 |
| TiHo12 | T | C | +0.484 | +0.170 |
| TiHo16 | T | C | +0.135 | -0.071 |
| TiHo18 | G* | T | +0.438 | -0.008 |
| TiHo19 | C | T | +0.919 | -0.197 |
| TiHo20 | C | T | +0.113 | +0.115 |
| TiHo21 | C | T | +0.793 | -0.158 |
| TiHo23 | C | T | +1.301 | +0.300 |
| TiHo24 | G | A | +0.522 | +0.070 |
| TiHo25 | G | A | +0.846 | +0.337 |
| TiHo26 | C | A | +0.671 | -0.287 |
| TiHo33 | A | G | +0.721 | -0.154 |
| TiHo34 | G | A | +0.227 | -0.184 |
| TiHo35 | G | C | +0.531 | +0.187 |

*: The CHD-associated allele was erroneously labelled ‘C’ in the patent specification owing to a confusion of the leading and lagging DNA strand.
