# Supplementary material for: Prospective evaluation of a patented DNA test for canine hip dysplasia (CHD)
Source: PLoS One. 2017 Aug 3;12(8):e0182093. doi: 10.1371/journal.pone.0182093 (PMC5542656; doi:10.1371/journal.pone.0182093)
Supplement: S1 Text — (PDF) [file pone.0182093.s004.pdf]

```
repl all score with 0

repl score with score-0.793 for .not.(TH21="N:N")
repl score with score+2*0.793 for TH21="C:C"
repl score with score+0.793-0.158 for .not.(left(TH21,1)=right(TH21,1))

repl score with score-1.301 for .not.(TH23="N:N")
repl score with score+2*1.301 for TH23="C:C"
repl score with score+1.301+0.300 for .not.(left(TH23,1)=right(TH23,1))

repl score with score-0.522 for .not.(TH24="N:N")
repl score with score+2*0.522 for TH24="G:G"
repl score with score+0.522+0.070 for .not.(left(TH24,1)=right(TH24,1))

repl score with score-0.846 for .not.(TH25="N:N")
repl score with score+2*0.846 for TH25="G:G"
repl score with score+0.846+0.337 for .not.(left(TH25,1)=right(TH25,1))

repl score with score-0.671 for .not.(TH26="N:N")
repl score with score+2*0.671 for TH26="C:C"
repl score with score+0.671-0.287 for .not.(left(TH26,1)=right(TH26,1))

repl score with score-0.721 for .not.(TH33="N:N")
repl score with score+2*0.721 for TH33="A:A"
repl score with score+0.721-0.154 for .not.(left(TH33,1)=right(TH33,1))

repl score with score-0.227 for .not.(TH34="N:N")
repl score with score+2*0.227 for TH34="G:G"
repl score with score+0.227-0.184 for .not.(left(TH34,1)=right(TH34,1))

repl score with score-0.531 for .not.(TH35="N:N")
repl score with score+2*0.531 for TH35="G:G"
repl score with score+0.531+0.187 for .not.(left(TH35,1)=right(TH35,1))

repl score with score-0.459 for .not.(TH01="N:N")
repl score with score+2*0.459 for TH01="G:G"
repl score with score+0.459+0.074 for .not.(left(TH01,1)=right(TH01,1))

repl score with score-0.477 for .not.(TH05="N:N")
repl score with score+2*0.477 for TH05="T:T"
repl score with score+0.477-0.036 for .not.(left(TH05,1)=right(TH05,1))

repl score with score-0.327 for .not.(TH07="N:N")
repl score with score+2*0.327 for TH07="T:T"
repl score with score+0.327+0.019 for .not.(left(TH07,1)=right(TH07,1))

repl score with score-0.391 for .not.(TH09="N:N")
repl score with score+2*0.391 for TH09="T:T"
repl score with score+0.391+0.016 for .not.(left(TH09,1)=right(TH09,1))

repl score with score-0.484 for .not.(TH12="N:N")
repl score with score+2*0.484 for TH12="T:T"
repl score with score+0.484+0.170 for .not.(left(TH12,1)=right(TH12,1))

repl score with score-0.135 for .not.(TH16="N:N")
repl score with score+2*0.135 for TH16="T:T"
repl score with score+0.135-0.071 for .not.(left(TH16,1)=right(TH16,1))

repl score with score-0.438 for .not.(TH18="N:N")
repl score with score+2*0.438 for TH18="G:G"
repl score with score+0.438-0.008 for .not.(left(TH18,1)=right(TH18,1))

repl score with score-0.919 for .not.(TH19="N:N")
```

```
repl score with score+2*0.919 for TH19="C:C"
repl score with score+0.919-0.197 for .not.(left(TH19,1)=right(TH19,1))

repl score with score-0.113 for .not.(TH20="N:N")
repl score with score+2*0.113 for TH20="C:C"
repl score with score+0.113+0.115 for .not.(left(TH20,1)=right(TH20,1))
```
